# Marine Oligo-Fucoidan as a Safe Functional Food for Managing Uterine Fibroids: Results from a Pilot Randomized Controlled Trial

**DOI:** 10.3390/biomedicines13081970

**Published:** 2025-08-13

**Authors:** Yi-Fen Chiang, Ko-Chieh Huang, Pei-Shen Huang, Mohamed Ali, Shih-Min Hsia

**Affiliations:** 1 School of Nutrition and Health Sciences, College of Nutrition, Taipei Medical University, Taipei 110301, Taiwan; yifenchiang@tmu.edu.tw (Y.-F.C.); da07111001@tmu.edu.tw (K.-C.H.); 2 Department of Obstetrics and Gynecology, Taipei Medical University Hospital, Taipei 110301, Taiwan; cannilyhuang@gmail.com; 3 Clinical Pharmacy Department, Faculty of Pharmacy, Ain Shams University, Cairo 11566, Egypt; mohamed.ali@bsd.uchicago.edu; 4 Department of Obstetrics and Gynecology, University of Chicago, Chicago, IL 60637, USA; 5 Graduate Institute of Metabolism and Obesity Sciences, College of Nutrition, Taipei Medical University, Taipei 110301, Taiwan; 6 School of Food Safety, Taipei Medical University, Taipei 110301, Taiwan; 7 Nutrition Research Center, Taipei Medical University Hospital, Taipei 110301, Taiwan; 8 TMU Research Center for Digestive Medicine, Taipei Medical University, Taipei 110301, Taiwan

**Keywords:** oligo-fucoidan, uterine leiomyoma, quality of life, randomized controlled trial, natural therapy

## Abstract

**Background:** Uterine leiomyomas, commonly known as fibroids, are the most prevalent benign tumors in women of reproductive age and a major contributor to gynecological morbidity. Although surgery and hormonal therapies are standard treatments, their associated side effects have prompted the search for safer, non-hormonal alternatives. Oligo-fucoidan, a sulfated polysaccharide derived from brown seaweed, has demonstrated anti-fibrotic and estrogen-regulating effects in preclinical models, but its clinical potential remains largely unexplored. **Methods:** In this randomized, double-blind, placebo-controlled pilot trial, 16 women diagnosed with uterine leiomyomas by ultrasound were enrolled and randomly assigned to receive either oligo-fucoidan (4 g/day) or placebo for six months (*n* = 8 per group). The primary endpoints were changes in the number of leiomyomas and quality of life, assessed using the World Health Organization Quality-of-Life Scale (WHOQOL-BREF) and Menstrual Distress Questionnaire (MDQ). Hormonal and safety parameters were also monitored. **Results:** Compared with the placebo group, participants receiving oligo-fucoidan exhibited a statistically significant reduction in fibroid number and reported improvements in quality-of-life domains. No serious adverse events occurred, and no clinically significant changes were noted in safety-related laboratory parameters. **Conclusions:** This pilot study provides preliminary clinical evidence that oligo-fucoidan may be a safe, well-tolerated, and potentially effective functional food-based approach for managing uterine fibroids. Larger trials are warranted to confirm these findings.

## 1. Introduction

Uterine leiomyomas, or fibroids, are the most common benign tumors of the female reproductive system and represent a significant public health concern, particularly among women of reproductive age. These monoclonal tumors originate from the smooth muscle cells of the myometrium and are characterized by aberrant extracellular matrix (ECM) accumulation, enhanced cell proliferation, and fibrotic remodeling [1]. While up to 70% of cases are asymptomatic, the remaining individuals often experience substantial clinical manifestations, including dysmenorrhea, menorrhagia, infertility, pregnancy loss, and chronic pelvic pain [2], which can severely impair quality of life (QOL) [3,4,5].

Conventional treatments, such as hormonal therapy, myomectomy, and hysterectomy, are effective but frequently associated with recurrence, adverse effects, and potential fertility loss. These limitations have driven increasing interest in alternative, non-hormonal interventions—particularly those derived from dietary or functional food sources—that may offer therapeutic benefits while minimizing systemic side effects [6,7].

Fucoidan, a sulfated polysaccharide abundant in brown seaweeds such as *Fucus vesiculosus* and *Undaria pinnatifida*, has emerged as a promising marine-derived functional compound with demonstrated anti-inflammatory, antioxidant, immunomodulatory, pain-relief and anti-fibrotic properties [8,9,10,11,12]. Preclinical evidence suggests that fucoidan modulates immune responses, inhibits ECM remodeling, and attenuates oxidative stress—mechanisms highly relevant to fibroid progression [13,14]. Our previous in vitro studies further indicate that oligo-fucoidan, a low-molecular-weight fucoidan, suppresses leiomyoma cell proliferation, induces apoptosis, and downregulates key fibrotic markers such as fibronectin, vimentin, α-smooth muscle actin (α-SMA), and Collagen 1A1 (COL1A1). Additionally, it inhibits SMAD family member 2/Extracellular signal-regulated kinase 1 and 2 (SMAD2/ERK1/2) phosphorylation and β-catenin nuclear translocation, thereby modulating the Transforming Growth Factor Beta 3 (TGF-β3) signaling axis [15,16,17].

Although preclinical data are promising, clinical evidence for oligo-fucoidan in uterine fibroids is limited. This study is the first randomized, double-blind, placebo-controlled trial to evaluate oligo-fucoidan’s effects on fibroid burden and quality of life in women with uterine leiomyomas. By addressing a critical gap in non-hormonal therapy, our findings may inform future dietary interventions and larger clinical trials.

## 2. Materials and Methods

### 2.1. Study Design and Ethical Approval

**Figure 1:**
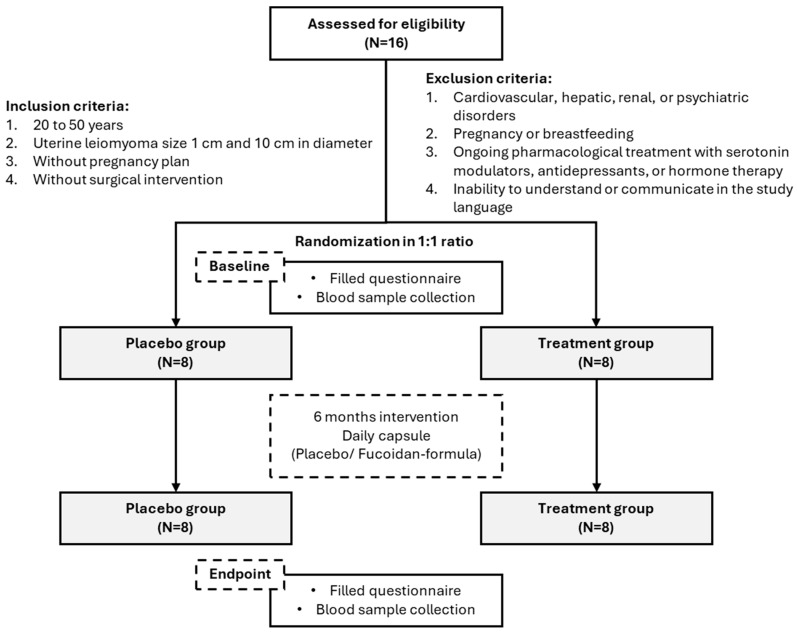
Study flowchart: Detailed description of all stages in this study. Flow chart illustrating the detailed process of participant enrollment, randomization, allocation to intervention or placebo groups, follow-up, and final analysis. All stages of the study, including screening, inclusion/exclusion, intervention duration, and outcome assessments, are presented.

This was a randomized, double-blind, placebo-controlled clinical trial conducted at Taipei Medical University Hospital to evaluate the effects of low-molecular-weight fucoidan (LMF, oligo-fucoidan) on the progression of uterine leiomyomas and symptom severity. The study included three phases: a screening period, a 24-week intervention phase, and final assessments at baseline and after 6 months. Participants were randomly assigned to receive either 4 g/day of oligo-fucoidan (treatment group) or a matched dose of cellulose placebo, administered in two divided doses daily. The LMF was extracted from Laminaria Japonica (*Saccharina japonica*) and manufactured by Hi-Q Marine Biotech International Ltd. (Taipei, Taiwan), as described previously [18] (Figure 1).

To maintain allocation concealment and ensure rigorous blinding, both active and placebo formulations were identical in appearance, packaging, and labeling. The random allocation sequence was generated and implemented independently, and the allocation was concealed from participants, investigators, care providers, and outcome assessors throughout the trial. Unblinding was only permitted after completion of data collection and database lock.

The full trial protocol, including statistical analysis plans, was reviewed and approved by the Taipei Medical University–Joint Institutional Review Board (TMU-JIRB; approval number: N201812056), ensuring compliance with ethical standards and research governance. The study was prospectively registered at ClinicalTrials.gov (Identifier: NCT06959966). All participants provided written informed consent prior to enrollment, in accordance with the principles of the Declaration of Helsinki. No protocol amendments were made during the study period. The finalized protocol and statistical plan are available from the corresponding author upon reasonable request.

### 2.2. Participant Recruitment and Eligibility Criteria

Participants were recruited through gynecology outpatient clinics at Taipei Medical University Hospital and targeted community advertisements. Eligible women were aged 20–50 years with ultrasound-confirmed uterine leiomyomas measuring 1–10 cm in diameter. Inclusion criteria required that participants were not pregnant, had no plans for pregnancy or surgical intervention during the study period, and were able and willing to comply with the study protocol. Importantly, only patients who had declined surgical treatment and were not receiving current hormonal or pharmacological therapy for leiomyomas were considered eligible.

Exclusion criteria included diagnosed cardiovascular, hepatic, renal, or psychiatric disorders; current pregnancy or breastfeeding; ongoing treatment with hormonal agents, antidepressants, or serotonin modulators; and inability to communicate fluently in the study language.

### 2.3. Biochemical Evaluation

Leiomyoma size was assessed by transabdominal and/or transvaginal ultrasonography at both baseline and the 6-month endpoint. Blood samples were collected at these time points, centrifuged at 4000 rpm for 10 min at 4 °C, and the serum was stored at –80 °C for subsequent biochemical analysis. Laboratory evaluations were performed by Asia Eastern Medical Laboratory (Taoyuan, Taiwan).

Hematological parameters included white blood cell (WBC) count, red blood cell (RBC) count, hemoglobin (HGB), hematocrit (Hct), neutrophils, lymphocytes, monocytes, basophils, eosinophils, platelets, mean corpuscular hemoglobin (MCH), mean corpuscular volume (MCV), and mean corpuscular hemoglobin concentration (MCHC). Biochemical assessments included fasting blood glucose, triglycerides (TG), total cholesterol (T-CHO), high-density lipoprotein cholesterol (HDL-C), low-density lipoprotein cholesterol (LDL-C), aspartate aminotransferase (AST), alanine aminotransferase (ALT), blood urea nitrogen (BUN), uric acid (UA), albumin (ALB), follicle-stimulating hormone (FSH), progesterone (PGT), vitamin D (VIT-D), androstenedione (ASD), and estradiol (E2). Systemic inflammation was evaluated using high-sensitivity C-reactive protein (HS-CRP).

### 2.4. Quality of Life Assessment (WHOQOL-BREF)

Quality of life was assessed using the validated Taiwan version of the WHOQOL-BREF instrument. This 28-item questionnaire encompasses four domains: physical health (7 items), psychological well-being (6 items), social relationships (4 items), and environmental factors (9 items), along with two general items measuring overall QOL and perceived general health. Responses were recorded on a 5-point Likert scale (1 = very poor; 5 = very good), allowing comprehensive evaluation of physical, emotional, social, and environmental dimensions of health-related quality of life (File S1).

### 2.5. Pain Type and Symptom Evaluation

Pain characteristics and severity were assessed using a structured pain questionnaire, covering multiple descriptors including throbbing, sharp, stabbing, cramping, gnawing, burning, persistent, dull, and tearing pain, as well as associated symptoms such as nausea, vomiting, and anxiety. Pain intensity was rated using a 4-point ordinal scale (0 = no pain; 3 = severe pain).

In addition, menstrual-related discomfort symptoms—including dizziness, muscle and breast pain, lower abdominal swelling, general body pain, back pain, headache, dermatologic issues, nausea, diarrhea, fatigue, weight gain, facial flushing, chills, and palpitations—were recorded and graded using a similar 4-point severity scale.

### 2.6. Statistical Analysis

All statistical analyses were conducted using GraphPad Prism 9.0 (GraphPad Software, San Diego, CA, USA). Data normality was confirmed using the Shapiro–Wilk test [19]. As all continuous variables followed a normal distribution, they were presented as means ± standard deviation (SD). Between-group comparisons were performed using independent (unpaired) t-tests, and within-group comparisons (before vs. after treatment) were analyzed using paired *t*-tests. A *p*-value < 0.05 was considered statistically significant.

To evaluate the statistical power post hoc, we conducted a power calculation using the ClinCalc Sample Size and Power Calculator (Kane SP, ClinCalc.com, updated 23 June 2024). Using a two-sided α of 0.05, the estimated power (1-β) was approximately 86.1% [20].

## 3. Results

### 3.1. Patient Characteristics at Baseline

Table 1 provides a summary of the baseline demographic and clinical characteristics of participants in the treatment and placebo groups. The average age of participants in the treatment group was 42.88 ± 3.64 years, while the average age in the placebo group was 44.88 ± 2.80 years, with no statistically significant difference between the two groups (*p* = 0.242). Other baseline characteristics, including age at menarche, height, weight, number of pregnancies, and number of deliveries, were also similar between the groups (all *p* > 0.05).

Regarding lifestyle factors, 12.5% of participants in the placebo group reported smoking, while none of the participants in the treatment group reported smoking. Both groups had similar menstrual cycle characteristics, with average cycle lengths of 29–31 days and menstrual durations of 4–5 days (*p* = 0.473 and *p* = 1.000, respectively).

At baseline, all participants in both the treatment and placebo groups had a confirmed diagnosis of uterine leiomyoma (100% in both groups). The prevalence of endometriosis was higher in the treatment group (37.5%) compared to the placebo group (12.5%), although this difference did not reach statistical significance (*p* = 0.156). Polycystic ovary syndrome (PCOS) was present in 12.5% of participants in the placebo group but was absent in the treatment group. Other conditions, including gynecological cancer, epilepsy, diabetes, and urinary tract infections (UTIs), were not reported in the treatment group, while 12.5% of participants in the placebo group reported a history of diabetes and UTIs.

No participants in either group reported a current pregnancy or plans for pregnancy within the next three months, ensuring a more homogenous baseline reproductive profile.

### 3.2. Blood Parameters and Biochemical Markers at Baseline and Endpoint

Hematological and biochemical parameters were evaluated at baseline and after the 6-month intervention to assess the potential systemic effects of oligo-fucoidan supplementation (Table 2). Overall, no significant differences were observed in most hematological parameters, including white blood cell (WBC) count, red blood cell (RBC) count, hemoglobin (HGB), hematocrit (Hct), differential white blood cell counts (neutrophils, lymphocytes, monocytes, basophils, eosinophils), platelet count, and red blood cell indices (mean corpuscular hemoglobin (MCH), mean corpuscular volume (MCV), and mean corpuscular hemoglobin concentration (MCHC)) between baseline and endpoint or between the treatment and placebo groups (all *p* > 0.05).

Similarly, common biochemical markers, including fasting blood glucose, lipid profile components (triglycerides (TG), total cholesterol (T-CHO), high-density lipoprotein cholesterol (HDL-C), low-density lipoprotein cholesterol (LDL-C)), inflammatory marker (high-sensitivity C-reactive protein (HS-CRP)), liver enzymes (glutamate oxaloacetate transaminase (GOT) and glutamate pyruvate transaminase (GPT)), kidney function markers (blood urea nitrogen (BUN) and uric acid (UA)), and albumin levels, showed no significant changes following treatment (all *p* > 0.05), highlighting the good safety profile of oligo-fucoidan.

One notable finding was a significant reduction in estradiol (E2) levels within both the treatment (151.13 ± 98.13 to 108.67 ± 54.61 pg/mL, *p* = 0.003) and placebo groups (140.14 ± 106.15 to 93.40 ± 76.00 pg/mL, *p* = 0.001) from baseline to endpoint. Despite these within-group reductions, no significant difference was observed between the treatment and placebo groups at either baseline (*p* = 0.81) or endpoint (*p* = 0.60), indicating that the observed E2 reductions were likely not attributable to the oligo-fucoidan intervention alone but possibly to natural hormonal variation or external factors.

Other hormonal markers, including follicle-stimulating hormone (FSH), progesterone (PGTR), vitamin D (VIT-D), and androstenedione (ASD), did not demonstrate significant changes within or between groups over time (all *p* > 0.05). This suggests that the oligo-fucoidan intervention had a minimal impact on broader endocrine parameters beyond E2.

Overall, the findings indicate that the oligo-fucoidan supplementation did not induce systemic alterations in hematologic, metabolic, or endocrine markers, apart from a non-specific reduction in E2 that warrants further investigation.

### 3.3. Impact of Fucoidan Formula on WHOQOL After 6-Month Intervention

Quality of life outcomes were assessed using the WHOQOL-BREF questionnaire (Table 3). The treatment group demonstrated a statistically significant improvement in total QOL scores from 2.75 ± 0.45 to 3.25 ± 0.45 (*p* = 0.026), while the placebo group showed no significant change. Between-group differences were significant at both baseline (*p* = 0.001) and endpoint (*p* = 0.026).

General health perception also differed significantly between groups at both time points, though no within-group change was observed. Physical health scores did not change significantly within either group but were higher in the treatment group at endpoint (*p* = 0.031). Psychological well-being improved in both groups, with a significant between-group difference at endpoint (*p* = 0.021). Social relationships and environmental domains remained unchanged. These findings indicate that oligo-fucoidan may positively influence QOL, particularly in the psychological domain, in women with uterine fibroids.

**Figure 2:**
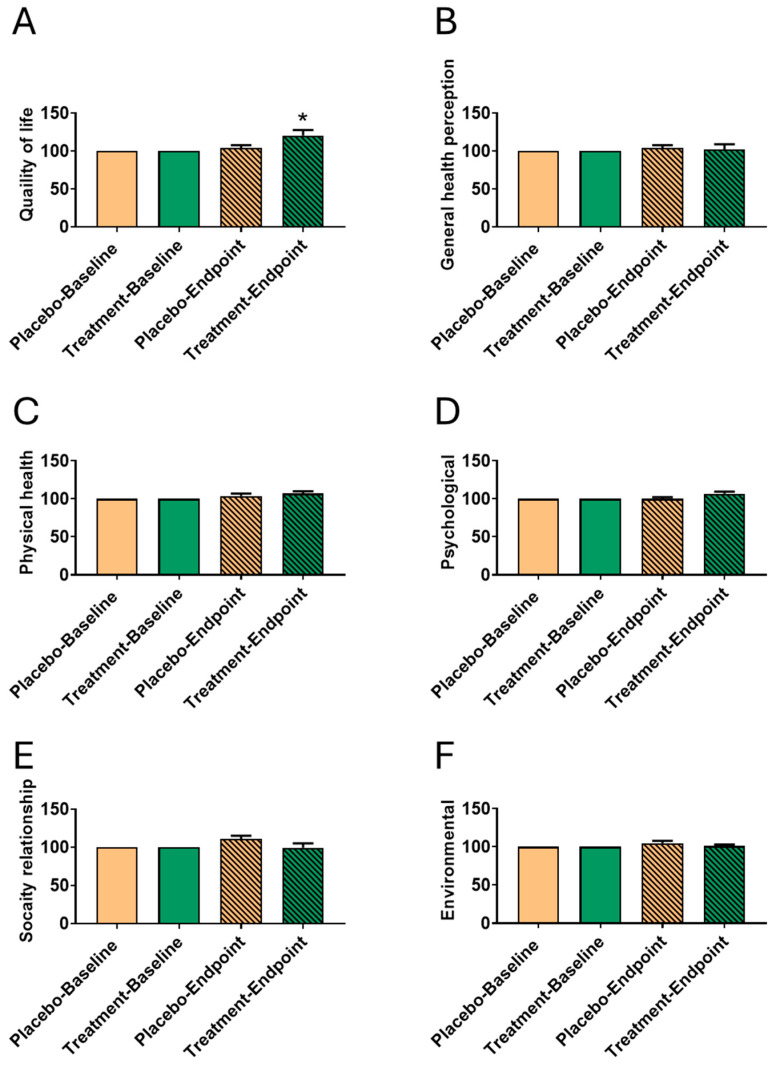
Baseline-normalized WHOQOL Scores before and after treatment. (**A**) Quality of life, (**B**) General health perception, (**C**) Physical health, (**D**) Psychological health, (**E**) Social relationships, and (**F**) Environmental health. All values were normalized to each individual’s baseline (set as 100%) to illustrate within-subject variability and treatment responsiveness. Data are presented as mean ± SD. *, *p* < 0.05 compared to the Treatment-baseline group.

To better illustrate individual variability and responsiveness, we have added a new figure presenting 100% baseline-normalized WHOQOL scores (Figure 2). This normalization highlights within-subject changes over time, accounting for baseline differences. The results support a trend of improvement in QOL following treatment. However, no additional statistically significant differences were observed beyond those previously reported.

### 3.4. Reduction in Leiomyoma Number

To evaluate the efficacy of the oligo-fucoidan formula in the reduction of leiomyoma burden, the number and size of leiomyomas were assessed in both the treatment and placebo groups at baseline and after the 6-month intervention (Table 4). To reduce the influence of inter-individual variability and improve the interpretability of the results, baseline values were normalized to 100%, allowing for a more accurate comparison of relative changes within each group.

**Figure 3:**
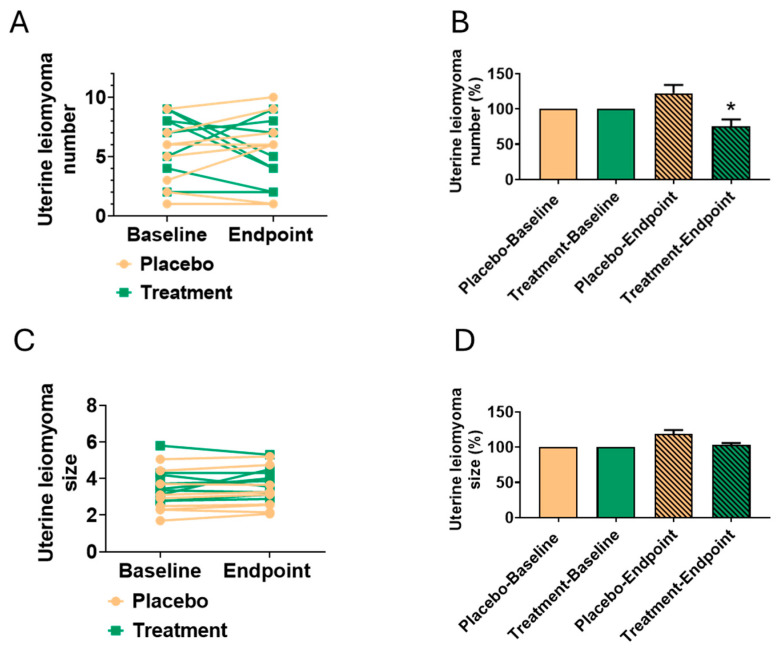
Effect of oligo-fucoidan formula on leiomyoma number reduction after a 6-month intervention. (**A**) Absolute change in leiomyoma count in the treatment and placebo groups over the 6-month intervention period. (**B**) Percentage change in leiomyoma counts relative to baseline, normalized to 100% to account for inter-individual variability. (**C**) Absolute size changes over the 6-month intervention period. (**D**) Percentage change in leiomyoma size relative to baseline, normalized to 100% to account for inter-individual variability. Data are expressed as mean ± SD, with statistical analysis performed using Student’s *t*-test. * *p* < 0.05 compared to baseline.

As illustrated in Figure 3A,C, the absolute number and size of leiomyomas in the treatment group over the 6-month intervention period decreased, although the magnitude of reduction varied among participants. This inter-individual variability likely reflects differences in baseline leiomyoma burden, disease progression rates, and individual responsiveness to the oligo-fucoidan intervention.

To further clarify the impact of the treatment, Figure 3B,D presents the percentage change in leiomyoma counts relative to baseline. This analysis revealed a consistent and statistically significant reduction in leiomyoma number, while the reduction in size showed a downward trend that did not reach statistical significance. In contrast, the placebo group exhibited no significant changes in either leiomyoma number or size.

These findings suggest that the reduction in leiomyoma number observed in the treatment group is likely attributable to the therapeutic effect of oligo-fucoidan, rather than to spontaneous regression or placebo response. While changes in size were not statistically significant, the observed trend warrants further investigation into larger and longer-term studies. Overall, the results support the potential of oligo-fucoidan as a non-hormonal therapeutic option for the reduction in leiomyoma burden, offering a potentially safer and more tolerable alternative to existing medical or surgical treatments.

### 3.5. Pain Type Assessment at Baseline and Endpoint

Pain is a common and often debilitating symptom in patients with uterine leiomyoma, manifesting in various forms, mainly cramping pain, as well as others, including stabbing, persistent, and burning pain. The assessment of pain types in this study revealed significant differences between the treatment and placebo groups over the 6-month intervention period (Table 5).

Cramping pain significantly increased in the placebo group from baseline to endpoint (*p* = 0.028), suggesting a potential progression of disease-related pain without intervention. In contrast, no significant change in cramping pain was observed in the treatment group (*p* = 0.785), indicating a possible stabilizing effect of the oligo-fucoidan formula. Notably, there was a significant difference in baseline cramping pain between the two groups (*p* = 0.008), reflecting variability in initial symptom severity.

Similarly, persistent pain significantly worsened in the placebo group (*p* = 0.031), with no corresponding deterioration in the treatment group, further supporting a possible protective effect of the oligo-fucoidan intervention. However, pain with touch decreased in the placebo group at the endpoint (*p* = 0.015), while no significant change was noted in the treatment group, potentially reflecting spontaneous symptom variation or a placebo effect.

Notably, stabbing pain showed a significant baseline difference between groups (*p* = 0.040), but no significant changes were noted within or between the groups at the endpoint, suggesting stability in this pain type over the study duration. Other pain modalities, including throbbing, sudden sharp, dull, and burning pain, did not exhibit statistically significant changes in either group, indicating overall stability in these symptoms regardless of intervention.

These findings suggest that the oligo-fucoidan formula may help prevent the worsening of certain pain types, particularly cramping and persistent pain, which appear more prone to progression in the absence of treatment. However, the variability in baseline pain scores and the modest sample size highlight the need for larger, long-term studies to confirm these preliminary observations and to better understand the potential of oligo-fucoidan in managing leiomyoma-associated pain.

### 3.6. Period Discomfort and Symptom Changes from Baseline to Endpoint in Treatment and Placebo Groups

The analysis of symptom severity across the treatment and placebo groups indicated no statistically significant differences within or between groups over the 6-month intervention period (Table 6). Symptom scores at baseline and endpoint remained comparable, with all *p*-values exceeding 0.05, indicating no meaningful changes in symptom severity over time.

Dizziness scores increased slightly in the treatment group from 1.50 ± 0.53 at baseline to 1.71 ± 0.76 at the endpoint (*p* = 0.705), while the placebo group showed minimal change (1.13 ± 0.35 to 1.14 ± 0.38, *p* = 1.000). Muscle pain also increased modestly in the treatment group (1.75 ± 0.71 to 2.14 ± 1.07, *p* = 0.809) and in the placebo group (1.50 ± 0.53 to 1.86 ± 0.69, *p* = 0.438), but neither change reached statistical significance.

Breast pain or swelling showed a slight increase in the treatment group (2.00 ± 0.76 to 2.57 ± 0.79, *p* = 0.619) and remained nearly stable in the placebo group (1.88 ± 0.83 to 1.86 ± 0.38, *p* = 0.717). Lower abdominal swelling exhibited a small increase in the treatment group (2.00 ± 0.76 to 2.43 ± 0.79, *p* = 0.798), while the placebo group experienced a minor decrease (2.50 ± 1.20 to 2.14 ± 0.90, *p* = 0.828), but neither trend was significant.

General body pain scores rose slightly in the treatment group (1.75 ± 0.89 to 2.00 ± 1.15, *p* = 1.000) and in the placebo group (1.50 ± 0.76 to 1.71 ± 0.76, *p* = 0.744), without significant differences. Back pain scores increased modestly in the treatment group (1.88 ± 0.64 to 2.14 ± 0.69, *p* = 1.000) and decreased slightly in the placebo group (2.25 ± 0.71 to 2.00 ± 0.82, *p* = 0.506), but these changes were also not statistically significant.

Headache severity remained stable in both groups, with scores of 1.75 ± 0.71 at baseline and 2.00 ± 1.15 at the endpoint in the treatment group (*p* = 1.000), and 2.38 ± 0.92 to 2.00 ± 1.00 in the placebo group (*p* = 0.672). Skin issues increased slightly in the treatment group (1.38 ± 1.06 to 1.71 ± 0.76, *p* = 0.805), while the placebo group showed a minor reduction (1.38 ± 0.52 to 1.14 ± 0.38, *p* = 0.619). Nausea or vomiting remained relatively stable in both groups, with minimal changes (treatment group: 1.00 ± 0.00 to 1.14 ± 0.38, *p* = 1.000; placebo group: 1.38 ± 0.52 to 1.14 ± 0.38, *p* = 0.278).

Diarrhea scores increased slightly in the treatment group (1.75 ± 1.16 to 2.29 ± 0.76, *p* = 1.000) and remained unchanged in the placebo group (2.62 ± 1.06 to 2.29 ± 0.76, *p* = 0.805). Fatigue showed minor fluctuations, decreasing slightly in the treatment group (2.38 ± 0.92 to 2.29 ± 0.76, *p* = 0.464) and in the placebo group (2.13 ± 0.99 to 1.71 ± 0.76, *p* = 0.227), but without significant differences. Weight gain scores declined modestly in the treatment group (1.50 ± 0.53 to 1.14 ± 0.38, *p* = 0.438), while remaining stable in the placebo group (1.13 ± 0.35 to 1.14 ± 0.38, *p* = 0.705).

Facial flushing increased slightly in the treatment group (1.00 ± 0.00 to 1.29 ± 0.49, *p* = 1.000) and remained nearly unchanged in the placebo group (1.13 ± 0.35 to 1.14 ± 0.38, *p* = 1.000). Chills showed a slight increase in the treatment group (1.25 ± 0.71 to 1.29 ± 0.49, *p* = 0.334) and remained stable in the placebo group (1.00 ± 0.00 to 1.00 ± 0.00, *p* = 1.000). Palpitations also exhibited minor changes in the treatment group (1.25 ± 0.71 to 1.29 ± 0.49, *p* = 0.717), while remaining unchanged in the placebo group (1.00 ± 0.00 to 1.00 ± 0.00).

**Figure 4:**
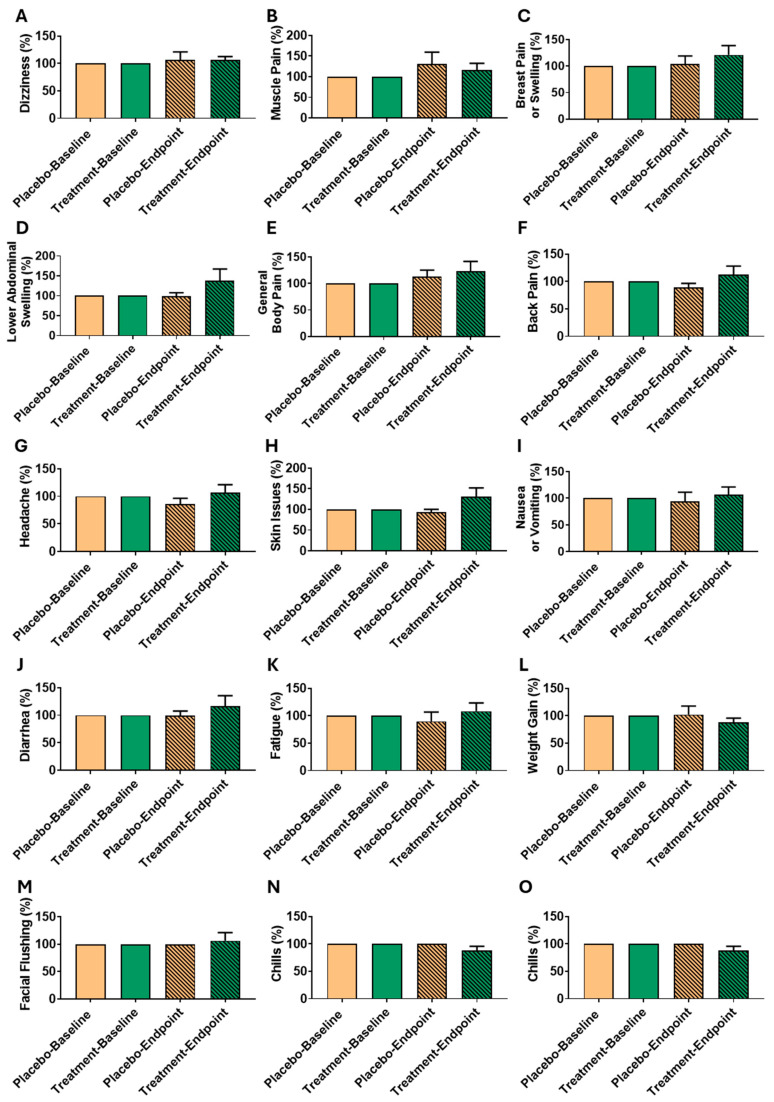
Baseline-normalized scores of discomfort symptoms before and after treatment. (**A**) Dizziness, (**B**) Muscle Pain, (**C**) Breast Pain or Swelling, (**D**) Lower Abdominal Swelling, (**E**) General Body Pain, (**F**) Back Pain, (**G**) Headache, (**H**) Skin Issues, (**I**) Nausea or Vomiting, (**J**) Diarrhea, (**K**) Fatigue, (**L**) Weight Gain, (**M**) Facial Flushing, (**N**) Chills, and (**O**) Palpitations. Each symptom score was normalized to the individual’s baseline (set as 100%) to reflect within-subject changes and variability. Data are presented as mean ± SD.

Given the subtle nature of these changes and to better explore intra-individual response variability, we also included a baseline-normalized comparison of symptom scores in Figure 4. This visualization demonstrates the percentage change relative to individual baselines, clarifying that while some participants showed subjective symptom improvement or worsening, these trends were inconsistent and did not yield statistically significant group-level changes.

Overall, none of the symptoms exhibited statistically significant changes within either group, nor were there significant differences between treatment and placebo groups at baseline or endpoint. These results suggest that while oligo-fucoidan may reduce leiomyoma burden, it does not exert broad symptomatic relief for general menstrual-related complaints during the study period. Further studies with larger sample sizes and longer follow-up durations are needed to confirm these preliminary findings and to explore the underlying mechanisms of oligo-fucoidan’s antifibrotic effects.

## 4. Discussion

This randomized pilot trial provides the first clinical evidence that oligo-fucoidan supplementation may reduce leiomyoma burden and improve quality of life in women with uterine fibroids. The observed reduction in leiomyoma number and improvement in WHOQOL-BREF scores suggest a potential role for oligo-fucoidan as a non-hormonal, functional food-based intervention.

Our findings are consistent with preclinical studies demonstrating anti-fibrotic and estrogen-modulating effects of fucoidan. Notably, the reduction in estradiol observed in both groups may reflect physiological variability rather than a treatment effect, highlighting the need for larger studies to clarify this relationship.

The observed trends in pain symptom changes should be interpreted with caution. Although some pain-related symptoms showed improvement in the treatment group, these findings were based on patient-reported outcomes using the Menstrual Distress Questionnaire (MDQ), which captures a broad spectrum of menstrual-related symptoms [21]. The reduction in certain pain descriptors may reflect the influence of the intervention on menstrual symptoms; however, due to the exploratory nature of this analysis and the subjective variability in symptom reporting, we have avoided making definitive claims about analgesic efficacy. While pain scores did not improve significantly, the positive impact on quality of life underscores the importance of patient-centered outcomes in fibroid management. These results support further investigation of oligo-fucoidan in larger, multicenter trials with longer follow-up.

The limited availability of effective, non-invasive, non-hormonal treatment options for uterine leiomyomas underscores the urgent need for alternative therapeutic strategies. Natural compounds, including oligo-fucoidan, have emerged as promising candidates due to their multi-targeted biological activities, which may simultaneously address symptom burden and structural progression of leiomyomas [22]. Previous studies have demonstrated the potential benefits of natural compounds in managing fibroid symptoms. For instance, vitamin D supplementation (50,000 IU for 12 weeks) has been shown to reduce disease progression in small uterine fibroids [23]. Similarly, Porcaro et al. (2020) reported that a combination of vitamin D, epigallocatechin-3-gallate (EGCG), and vitamin B6 reduced fibroid volume by 34.7% compared to 6.9% in the placebo group, suggesting a potential role for these compounds in non-hormonal uterine fibroid management [24]. Other studies have highlighted the synergistic effects of vitamin D with ulipristal acetate (UPA) in reducing fibroid volume, further supporting the therapeutic potential of combining natural compounds for fibroid management [25,26,27,28].

In addition to these findings, EGCG, a polyphenol found in green tea, has demonstrated significant anti-inflammatory, antioxidant, and anti-proliferative effects in preclinical and clinical studies [29,30,31]. Daily supplementation with 800 mg of tea extract containing 45% EGCG for 4 months reduced fibroid volume by 32.6% and improved associated symptoms, including menstrual bleeding and anemia [32]. These studies collectively indicate that natural compounds may modulate key signaling pathways involved in fibroid pathogenesis, supporting their potential for broader clinical application.

One intriguing finding from the current study was the significant reduction in estradiol (E2) levels in both the treatment and placebo groups. This is particularly relevant given the well-established role of estrogen in promoting leiomyoma growth. Gonadotropin-releasing hormone (GnRH) agonists, a mainstay in medical management of uterine leiomyomas, exert their effects primarily by inducing a hypoestrogenic state [33]. Although the exact mechanism by which oligo-fucoidan might influence estrogen levels remains unclear, prior studies have suggested that oligo-fucoidan may impact estrogen-dependent pathways. For example, oligo-fucoidan has been shown to reduce E2 levels in premenopausal women with endometriosis, potentially through modulation of estrogen metabolism or receptor signaling [34,35].

However, the observed E2 reduction in the placebo group was unexpected and may reflect underlying physiological variability. Estradiol homeostasis is tightly regulated and can be influenced by multiple factors, including stress, lifestyle changes, seasonal variation, diet, physical activity, and spontaneous hormonal fluctuations [36,37,38]. To minimize hormonal variation related to the menstrual cycle, participants were instructed to avoid sample collection during their menstrual period, and sampling was scheduled during the mid-luteal phase whenever feasible. However, given the small sample size of this pilot study, random variability in hormone levels could still have contributed to the observed results. It is also possible that lifestyle changes during the study period, such as altered sleep or stress levels, may have influenced E2 levels. Larger-scale studies with more rigorous hormonal timing and control of external factors are necessary to determine whether the E2 reduction observed in the treatment group reflects a true biological effect of oligo-fucoidan or a coincidental finding.

In addition to its potential effects on estrogen levels, the oligo-fucoidan intervention significantly improved QOL in the treatment group, as measured by the WHOQOL-BREF instrument. This improvement, despite the absence of significant pain reduction, suggests that oligo-fucoidan may positively influence broader aspects of patient well-being, including physical and psychological health. Given that QOL is a critical outcome in the management of chronic gynecological conditions like leiomyomas, this finding supports the potential role of oligo-fucoidan as an adjunctive treatment to improve overall patient outcomes [39].

### Study Limitations

This study has several limitations. First, the relatively short duration of the intervention (6 months) may have been insufficient to capture the full therapeutic potential of oligo-fucoidan, particularly in terms of pain reduction and long-term fibroid shrinkage. Uterine leiomyomas are chronic conditions, and longer treatment durations may be required to observe more substantial symptom relief. Second, the small sample size limits the generalizability of the findings and increases the potential for random variability in hormone levels, as noted with the unexpected E2 reductions in the placebo group. Third, the study population was recruited from a single center, which may introduce selection bias. Fourth, while randomization and blinding were implemented, residual confounding from unmeasured variables cannot be excluded. Future multicenter studies with larger sample sizes and longer follow-up are warranted to confirm these preliminary results and further elucidate the mechanisms of action.

Moreover, bioavailability remains a significant challenge for fucoidan and other dietary phytochemicals. Like many natural compounds, fucoidan may suffer from poor absorption, rapid metabolism, and limited tissue distribution, potentially compromising its therapeutic efficacy [40,41]. Strategies to enhance bioavailability, including nanoparticle delivery systems, improved extraction methods, and combination therapies, may improve the clinical utility of fucoidan in future studies.

Finally, the heterogeneity of the study population and the lack of precise biomarker assessments may have further contributed to the variability in treatment responses. Future studies should consider stratifying participants based on fibroid size, hormonal profiles, and baseline symptom severity to better identify responders and optimize therapeutic outcomes.

Despite these limitations, to our knowledge, this is the first randomized controlled trial evaluating oligo-fucoidan as a non-hormonal intervention for uterine leiomyomas. The findings from this pilot study provide preliminary evidence that oligo-fucoidan may reduce leiomyoma burden and improve QOL in affected women. These results underscore the need for larger, well-designed clinical trials to confirm the therapeutic potential of oligo-fucoidan, explore its mechanisms of action, and optimize its clinical application through improved formulation and targeted delivery strategies. Continued investigation into the pharmacokinetics, maximum dosing, and potential synergistic effects of oligo-fucoidan with other natural compounds may further enhance its therapeutic profile in the management of uterine leiomyomas.

## 5. Conclusions

In conclusion, this pilot study suggests that oligo-fucoidan supplementation may reduce the number of uterine leiomyomas and improve quality of life in affected women, supporting its potential as a promising non-hormonal, functional food-derived intervention. Although no significant improvement in pain was observed, the reductions in fibroid burden and enhancement in QOL highlight oligo-fucoidan’s promise as a safe adjunctive therapy for fibroid management. However, the observed decline in estradiol levels in both treatment and placebo groups, along with the lack of significant changes in other hormonal or inflammatory markers, underscores the complexity of fibroid pathophysiology and the impact of individual variability. To confirm these findings, larger multicenter trials with longer follow-up, stratified analyses, and mechanistic exploration are warranted. Additionally, improving the bioavailability of fucoidan will be essential for realizing its full therapeutic potential. Innovations in delivery systems and synergistic combinations with other functional compounds may enhance absorption, target engagement, and clinical efficacy—ultimately advancing oligo-fucoidan as an evidence-based nutraceutical strategy for managing uterine leiomyomas.

## Figures and Tables

**Table 1:** Baseline demographic and clinical characteristics of participants in the treatment and placebo groups.

Characteristic/Condition	Treatment Group (*n* = 8)	Placebo Group (*n* = 8)	*p*-Value
Age (years)	42.88 ± 3.64	44.88 ± 2.80	0.242
Menarche age (years)	10.25 ± 6.39	11.00 ± 4.72	0.794
Height (cm)	158.88 ± 4.70	158.50 ± 2.93	0.851
Weight (kg)	55.00 ± 10.25	54.88 ± 6.92	0.978
Number of pregnancies	0.88 ± 1.25	0.50 ± 0.76	0.559
Number of deliveries	0.63 ± 0.92	0.38 ± 0.74	0.473
Smoking (n, %)	0 (0%)	1 (12.5%)	-
Average cycle length (days)	29–31	29–31	0.473
Menstrual duration (days)	4–5	4–5	1.000
First dysmenorrhea onset	After menarche (>3 years)	After menarche (>3 years)	0.737
Dysmenorrhea timing	1–3 days during menstruation	1–3 days during menstruation	0.662
Dysmenorrhea persistence (days)	1–2 days	2–3 days	0.324
Impact severity of dysmenorrhea	Mild to moderate	Mild to moderate	0.849
Uterine Leiomyoma	8/8 (100%)	8/8 (100%)	-
Endometriosis	3/8 (37.5%)	1/8 (12.5%)	-
PCOS	0/8 (0%)	1/8 (12.5%)	-
Gynecological Cancer	0/8 (0%)	0/8 (0%)	-
Epilepsy	0/8 (0%)	0/8 (0%)	-
Diabetes	0/8 (0%)	1/8 (12.5%)	-
UTI	0/8 (0%)	1/8 (12.5%)	-
Pregnancy	0/8 (0%)	0/8 (0%)	-
Pregnancy Plan in 3 Months	0/8 (0%)	0/8 (0%)	-

Footnote: Pregnancy and delivery (times): 0 = None; Smoking: 0 = No; Average cycle length: 1 = ≤28 days, 2 = 29–31 days, 3 = 32–34 days, 4 = 35–38 days, 5 = 39–41 days, 6 = Irregular. Menstrual duration: 2–3 days, 4–5 days, 6–7 days, ≥8 days; First dysmenorrhea onset: At menarche, 6 months–3 years after menarche, >3 years after menarche, Forgot, Other. Dysmenorrhea timing: One week before menstruation, 1–3 days before menstruation, 1–3 days during menstruation, One week during menstruation, After menstruation, Other. Dysmenorrhea persistence: 1 day, 2 days, 3 days, >3 days. Impact severity of dysmenorrhea: None, Mild, Moderate, Severe. Polycystic ovary syndrome (PCOS); urinary tract infections (UTI).

**Table 2:** Blood parameters and biochemical markers at baseline and endpoint in treatment and placebo groups.

Parameter (Unit)	Mean ± SD				*p*-Value			
	Treatment		Placebo		Baseline vs. Endpoint		Treatment vs. Placebo	
	Baseline	Endpoint	Baseline	Endpoint	Treatment	Placebo	Baseline	Endpoint
WBC 10^3^/μL)	6.21 ± 1.47	7.48 ± 2.61	5.11 ± 1.08	6.06 ± 1.90	0.999	0.999	0.999	0.999
RBC (10^6^/μL)	4.23 ± 0.33	4.25 ± 0.30	4.25 ± 0.53	4.37 ± 0.55	0.999	0.999	0.999	0.999
HGB (g/dL)	12.45 ± 1.65	13.02 ± 0.88	12.13 ± 1.49	11.46 ± 2.73	0.999	0.999	0.999	0.999
Hct (%)	37.78 ± 4.72	38.53 ± 2.28	36.81 ± 3.90	35.66 ± 6.26	0.999	0.999	0.999	0.999
Net-s (%)	61.24 ± 6.25	58.00 ± 11.24	47.66 ± 18.73	58.76 ± 10.26	0.999	0.814	0.692	0.999
Lym-L (%)	26.36 ± 6.27	29.48 ± 8.13	39.25 ± 11.94	31.74 ± 9.77	0.999	0.932	0.724	0.999
Mono (%)	9.14 ± 1.97	8.85 ± 2.39	8.73 ± 4.81	6.48 ± 2.01	0.999	0.999	0.999	0.999
Baso (%)	0.84 ± 0.24	0.82 ± 0.31	0.93 ± 0.44	1.00 ± 0.45	0.999	0.999	0.999	0.999
Eosin (%)	2.43 ± 1.51	2.85 ± 3.19	3.44 ± 3.66	2.02 ± 1.55	0.999	0.999	0.999	0.999
Platel (10^3^/μL)	313.63 ± 71.77	314.17 ± 99.72	281.13 ± 71.55	304.20 ± 68.67	0.999	0.242	0.999	0.852
MCH (pg)	29.30 ± 2.32	30.67 ± 1.11	28.31 ± 3.89	26.38 ± 5.79	0.999	0.999	0.999	0.999
MCV (fL)	89.10 ± 6.32	90.75 ± 2.18	85.66 ± 9.57	82.14 ± 13.18	0.999	0.999	0.999	0.909
MCHC (g/dL)	32.89 ± 0.85	33.80 ± 0.60	32.93 ± 1.31	31.78 ± 2.43	0.999	0.999	0.999	0.999
Glucose (mg/dL)	83.88 ± 3.98	83.67 ± 5.61	93.88 ± 15.72	93.20 ± 12.44	0.999	0.999	0.852	0.863
TG (mg/dL)	72.13 ± 21.33	76.83 ± 21.42	85.50 ± 28.19	94.00 ± 41.79	0.983	0.901	0.703	0.512
T-CHO (mg/dL)	181.75 ± 29.90	206.00 ± 34.17	183.00 ± 40.80	187.20 ± 49.82	0.200	0.987	0.999	0.423
HDL-C (mg/dL)	65.13 ± 16.99	66.33 ± 11.69	66.00 ± 11.03	61.60 ± 18.85	0.999	0.999	0.999	0.999
LDL-C (mg/dL)	108.75 ± 23.71	129.00 ± 28.71	108.63 ± 41.73	120.60 ± 39.45	0.352	0.773	0.999	0.902
HS-CRP (mg/dL)	2.45 ± 2.76	1.89 ± 3.09	1.16 ± 1.44	1.10 ± 1.76	0.999	0.999	0.999	0.999
GOT (U/L)	18.25 ± 5.26	18.50 ± 6.09	17.63 ± 3.81	18.20 ± 3.19	0.999	0.999	0.999	0.999
GPT (U/L)	16.00 ± 8.35	14.50 ± 4.28	15.25 ± 4.68	12.40 ± 1.82	0.999	0.999	0.999	0.999
BUN (mg/dL)	11.63 ± 2.62	12.33 ± 1.86	12.50 ± 2.62	11.40 ± 2.51	0.999	0.999	0.999	0.999
UA (mg/dL)	4.50 ± 1.31	4.68 ± 0.94	4.43 ± 0.96	4.30 ± 1.00	0.999	0.999	0.999	0.999
ALB (g/dL)	4.23 ± 0.25	4.27 ± 0.15	4.30 ± 0.17	4.42 ± 0.13	0.999	0.999	0.999	0.999
FSH (mIU/mL)	6.58 ± 3.04	17.91 ± 23.80	10.05 ± 11.24	6.50 ± 2.94	0.794	0.999	0.999	0.792
PGTR (ng/mL)	5.41 ± 3.89	3.35 ± 3.37	7.01 ± 7.95	9.67 ± 12.70	0.999	0.999	0.999	0.999
VIT-D (ng/mL)	22.09 ± 13.66	22.20 ± 8.20	21.74 ± 5.97	23.70 ± 1.52	0.999	0.999	0.999	0.999
ASD (ng/mL)	1.10 ± 0.63	1.29 ± 0.71	0.94 ± 0.46	0.69 ± 0.28	0.999	0.999	0.999	0.999
E2 (pg/mL)	151.13 ± 98.13	108.67 ± 54.61	140.14 ± 106.15	93.40 ± 76.00	0.003 *	0.001 *	0.812	0.602

WBC (White Blood Cell Count); RBC (Red Blood Cell Count); HGB (Hemoglobin); Hct (Hematocrit); Net-s (Neutrophil percentage); Lym-L (Lymphocyte percentage); Mono (Monocyte percentage); Baso (Basophil percentage); Eosin (Eosinophil percentage); Platel (Platelet Count); MCH (Mean Corpuscular Hemoglobin); MCV (Mean Corpuscular Volume); MCHC (Mean Corpuscular Hemoglobin Concentration); TG (Triglycerides); T-CHO (Total Cholesterol); HDL-c (High-Density Lipoprotein Cholesterol); LDL-C (Low-Density Lipoprotein Cholesterol); HS-CRP (High-Sensitivity C-Reactive Protein); GOT (Glutamate Oxaloacetate Transaminase); GPT (Glutamate Pyruvate Transaminase); BUN (Blood Urea Nitrogen); UA (Uric Acid); ALB (Albumin); FSH (Follicle-Stimulating Hormone); PGTR (Progesterone); VIT-D (Vitamin D); ASD (Androstenedione); E2 (Estradiol). *, *p* < 0.05, statistically significant difference.

**Table 3:** Effect of fucoidan formula on WHOQOL scores after a 6-month intervention.

	Mean ± SD				*p*-Value			
WHOQOL-BREF	Treatment		Placebo		Baseline vs. Endpoint		Treatment vs. Placebo	
	Baseline	Endpoint	Baseline	Endpoint	Treatment	Placebo	Baseline	Endpoint
Quality of life	2.75 ± 0.45	3.25 ± 0.45	3.63 ± 0.50	3.75 ± 0.45	0.026 *	0.572	0.001 *	0.026 *
General health perception	2.50 ± 0.73	2.50 ± 0.73	3.50 ± 0.50	3.63 ± 0.50	1.000	0.597	0.003 *	0.001 *
Physical health	2.98 ± 0.42	2.98 ± 0.35	3.20 ± 0.38	3.36 ± 0.25	1.000	0.354	0.303	0.031 *
Psychological	3.06 ± 0.23	3.17 ± 0.42	3.40 ± 0.42	3.60 ± 0.33	0.503	0.274	0.062	0.021 *
Social relationships	3.51 ± 0.42	3.43 ± 0.55	3.47 ± 0.47	3.81 ± 0.39	0.758	0.106	0.881	0.086
Environmental	3.74 ± 0.47	3.61 ± 0.48	3.83 ± 0.40	3.99 ± 0.39	0.580	0.458	0.669	0.078

*, *p* < 0.05, statistically significant difference.

**Table 4:** Leiomyoma number and size in the treatment and placebo groups at baseline and endpoint.

	Treatment		Placebo		Baseline vs. Endpoint		Treatment vs. Placebo	
Leiomyoma Tissue	Baseline	Endpoint	Baseline	Endpoint	Treatment	Placebo	Baseline	Endpoint
Number	6.00 ± 3.00	4.78 ± 2.31	4.44 ± 2.87	5.22 ± 3.28	0.361	0.609	0.261	0.764
Size	3.72 ± 1.02	3.86 ± 0.82	3.11 ± 1.17	3.26 ± 1.18	0.757	0.801	0.286	0.254

**Table 5:** Pain type assessment in the treatment and placebo groups at baseline and endpoint.

	Mean ± SD				*p*-Value			
Pain Type	Treatment		Placebo		Baseline vs. Endpoint		Treatment vs. Placebo	
	Baseline	Endpoint	Baseline	Endpoint	Treatment	Placebo	Baseline	Endpoint
Throbbing Pain	0.75 ± 0.71	0.86 ± 0.90	0.63 ± 0.52	1.14 ± 0.90	0.800	0.187	0.692	0.563
Sudden Sharp Pain	0.38 ± 0.74	0.71 ± 0.76	0.50 ± 0.53	0.86 ± 1.07	0.397	0.418	0.705	0.777
Stabbing Pain	0.13 ± 0.35	0.43 ± 0.79	0.63 ± 0.52	0.57 ± 0.79	0.341	0.894	0.040 *	0.768
Cutting Pain	0.13 ± 0.35	0.14 ± 0.38	0.00 ± 0.00	0.43 ± 0.79	0.926	0.145	0.334	0.403
Cramping Pain	1.00 ± 0.93	1.14 ± 1.07	0.63 ± 1.19	1.00 ± 1.15	0.785	0.028 *	0.008 *	0.814
Gnawing Pain	0.00 ± 0.00	0.29 ± 0.49	0.63 ± 1.18	0.57 ± 0.79	0.119	0.930	0.158	0.551
Burning Pain	0.13 ± 0.35	0.29 ± 0.49	0.00 ± 0.00	1.00 ± 1.07	0.598	0.028 *	0.333	0.196
Persistent Pain	0.50 ± 0.76	0.57 ± 0.72	0.25 ± 0.46	1.29 ± 0.76	0.838	0.031 *	0.505	0.108
Dull Pain	1.13 ± 0.64	1.43 ± 0.62	1.25 ± 0.46	1.14 ± 0.38	0.424	0.856	0.808	0.539
Pain with Touch	0.00 ± 0.00	0.00 ± 0.00	1.63 ± 0.74	0.29 ± 0.53	-	0.015 *	0.001 *	0.147
Tearing Pain	0.13 ± 0.35	0.57 ± 0.71	0.00 ± 0.00	0.43 ± 0.79	0.368	0.145	0.334	0.175
Exhaustion Feeling	0.25 ± 0.46	0.57 ± 0.79	0.50 ± 0.53	0.71 ± 0.95	0.473	0.052	0.334	0.786
Nausea/Vomiting Feeling	0.50 ± 0.71	0.00 ± 0.00	0.50 ± 0.53	0.86 ± 0.98	0.838	0.418	1.000	0.299
Fear/Anxiety Feeling	0.25 ± 0.46	0.00 ± 0.00	0.50 ± 0.53	0.43 ± 0.79	0.368	0.532	0.505	0.147
Excruciating Pain	0.00 ± 0.00	0.00 ± 0.00	0.00 ± 0.00	0.43 ± 0.79	-	0.145	-	0.175

*, *p* < 0.05, statistically significant difference.

**Table 6:** Discomfort symptom changes from baseline to endpoint in treatment and placebo groups.

Symptom	Mean ± SD				*p*-Value			
	Treatment		Placebo		Baseline vs. Endpoint		Treatment vs. Placebo	
	Baseline	Endpoint	Baseline	Endpoint	Treatment	Placebo	Baseline	Endpoint
Dizziness	1.50 ± 0.53	1.71 ± 0.76	1.13 ± 0.35	1.14 ± 0.38	0.705	1.000	0.12	0.108
Muscle Pain	1.75 ± 0.71	2.14 ± 1.07	1.50 ± 0.53	1.86 ± 0.69	0.809	0.438	0.438	0.809
Breast Pain or Swelling	2.00 ± 0.76	2.57 ± 0.79	1.88 ± 0.83	1.86 ± 0.38	0.619	0.717	0.758	0.278
Lower Abdominal Swelling	2.00 ± 0.76	2.43 ± 0.79	2.50 ± 1.20	2.14 ± 0.90	0.798	0.828	0.334	0.655
General Body Pain	1.75 ± 0.89	2.00 ± 1.15	1.50 ± 0.76	1.71 ± 0.76	1.000	0.744	0.554	0.815
Back Pain	1.88 ± 0.64	2.14 ± 0.69	2.25 ± 0.71	2.00 ± 0.82	1.000	0.506	0.285	0.781
Headache	1.75 ± 0.71	2.00 ± 1.15	2.38 ± 0.92	2.00 ± 1.00	1.000	0.672	0.149	0.579
Skin Issues	1.38 ± 1.06	1.71 ± 0.76	1.38 ± 0.52	1.14 ± 0.38	0.805	0.619	1.000	0.506
Nausea or Vomiting	1.00 ± 0.00	1.14 ± 0.38	1.38 ± 0.52	1.14 ± 0.38	1.000	0.278	0.060	0.590
Diarrhea	1.75 ± 1.16	2.29 ± 0.76	2.62 ± 1.06	2.29 ± 0.76	1.000	0.805	0.139	0.176
Fatigue	2.38 ± 0.92	2.29 ± 0.76	2.13 ± 0.99	1.71 ± 0.76	0.464	0.227	0.609	0.387
Weight Gain	1.50 ± 0.53	1.14 ± 0.38	1.13 ± 0.35	1.14 ± 0.38	0.438	0.705	1.000	0.693
Facial Flushing	1.00 ± 0.00	1.29 ± 0.49	1.13 ± 0.35	1.14 ± 0.38	1.000	1.000	0.334	0.590
Chills	1.25 ± 0.71	1.29 ± 0.49	1.00 ± 0.00	1.00 ± 0.00	0.334	1.000	0.554	0.590
Palpitations	1.25 ± 0.71	1.29 ± 0.49	1.00 ± 0.00	1.00 ± 0.00	0.717	-	0.334	0.590

## Data Availability

Data are available on request due to privacy restrictions.

## Supplementary Materials

The following supporting information can be downloaded at: https://www.mdpi.com/article/10.3390/biomedicines13081970/s1, File S1: Taiwanese WHOQOL-BREF.

## Abbreviations

The following abbreviations are used in this manuscript:

ALB	Albumin
ASD	Androstenedione
α-SMA	Alpha-Smooth Muscle Actin
Baso	Basophil Percentage
BUN	Blood Urea Nitrogen
COL1A1	Collagen Type I Alpha 1 Chain
E2	Estradiol
ECM	Extracellular Matrix
EGCG	Epigallocatechin-3-Gallate
Eosin	Eosinophil Percentage
ERK1/2	Extracellular Signal-Regulated Kinase 1/2
FSH	Follicle-Stimulating Hormone
GPT	Glutamate Pyruvate Transaminase
GOT	Glutamate Oxaloacetate Transaminase
HDL-C	High-Density Lipoprotein Cholesterol
Hct	Hematocrit
HGB	Hemoglobin
HS-CRP	High-Sensitivity C-Reactive Protein
LDL-C	Low-Density Lipoprotein Cholesterol
Lym-L	Lymphocyte Percentage
MCH	Mean Corpuscular Hemoglobin
MCHC	Mean Corpuscular Hemoglobin Concentration
MCV	Mean Corpuscular Volume
Mono	Monocyte Percentage
Net-s	Neutrophil Percentage
PCOS	Polycystic Ovary Syndrome
PGTR	Progesterone
Platel	Platelet Count
QOL	Quality of Life
RBC	Red Blood Cell Count
Smad2	SMAD Family Member 2
T-CHO	Total Cholesterol
TG	Triglycerides
UA	Uric Acid
UTI	Urinary Tract Infection
VIT-D	Vitamin D
WBC	White Blood Cell Count
