# Supplementary material for: Marine Oligo-Fucoidan as a Safe Functional Food for Managing Uterine Fibroids: Results from a Pilot Randomized Controlled Trial
Source: Biomedicines. 2025 Aug 13;13(8):1970. doi: 10.3390/biomedicines13081970 (PMC12383344; doi:10.3390/biomedicines13081970)
Supplement: Supplementary file 1 [file biomedicines-13-01970-s001.zip › biomedicines-3778718-supplementary.pdf]

**Taiwanese WHOQOL-BREF**

1. Overall, how would you rate your quality of life?
   ☐ Very poor ☐ Poor ☐ Neither poor nor good ☐ Good ☐ Very good
2. Overall, how satisfied are you with your health?
   ☐ Very dissatisfied ☐ Dissatisfied ☐ Neither satisfied nor dissatisfied ☐ Satisfied ☐ Very satisfied
3. To what extent do you feel physical pain prevents you from doing what you need to do?
   ☐ Not at all ☐ A little ☐ A moderate amount ☐ Very much ☐ An extreme amount
4. How much do you need medical treatment to function in your daily life?
   ☐ Not at all ☐ A little ☐ A moderate amount ☐ Very much ☐ An extreme amount
5. Do you enjoy your life?
   ☐ Not at all ☐ A little ☐ A moderate amount ☐ Very much ☐ An extreme amount
6. To what extent do you feel your life to be meaningful?
   ☐ Not at all ☐ A little ☐ A moderate amount ☐ Very much ☐ An extreme amount
7. How well are you able to concentrate (e.g., thinking, learning, remembering)?
   ☐ Very poor ☐ Poor ☐ Moderate ☐ Good ☐ Very good
8. How safe do you feel in your daily life?
   ☐ Very unsafe ☐ Unsafe ☐ Moderately safe ☐ Safe ☐ Very safe
9. How healthy is your physical environment? (e.g., pollution, noise, climate, scenery)
   ☐ Very unhealthy ☐ Unhealthy ☐ Moderately healthy ☐ Healthy ☐ Very healthy
10. Do you have enough energy for daily life?
    ☐ Not at all ☐ A little ☐ A moderate amount ☐ Very much ☐ Completely
11. Are you able to accept your appearance?
    ☐ Not at all ☐ A little ☐ A moderate amount ☐ Very much ☐ Completely
12. Do you have enough money to meet your needs?
    ☐ Not at all ☐ A little ☐ A moderate amount ☐ Very much ☐ Completely
13. How available is the information you need in your day-to-day life?
    ☐ Not at all ☐ A little ☐ A moderate amount ☐ Very much ☐ Completely
14. Do you have opportunities for leisure activities?
    ☐ Not at all ☐ A little ☐ A moderate amount ☐ Very much ☐ Completely

15. How well are you able to get around?
☐ Very poor ☐ Poor ☐ Moderate ☐ Good ☐ Very good
16. How satisfied are you with your sleep?
☐ Very dissatisfied ☐ Dissatisfied ☐ Neither satisfied nor dissatisfied ☐ Satisfied ☐ Very satisfied
17. How satisfied are you with your ability to perform daily living activities?
☐ Very dissatisfied ☐ Dissatisfied ☐ Neither satisfied nor dissatisfied ☐ Satisfied ☐ Very satisfied
18. How satisfied are you with your capacity for work?
☐ Very dissatisfied ☐ Dissatisfied ☐ Neither satisfied nor dissatisfied ☐ Satisfied ☐ Very satisfied
19. How satisfied are you with yourself?
☐ Very dissatisfied ☐ Dissatisfied ☐ Neither satisfied nor dissatisfied ☐ Satisfied ☐ Very satisfied
20. How satisfied are you with your personal relationships?
☐ Very dissatisfied ☐ Dissatisfied ☐ Neither satisfied nor dissatisfied ☐ Satisfied ☐ Very satisfied
21. How satisfied are you with your sexual life?
☐ Very dissatisfied ☐ Dissatisfied ☐ Neither satisfied nor dissatisfied ☐ Satisfied ☐ Very satisfied
22. How satisfied are you with the support you get from your friends?
☐ Very dissatisfied ☐ Dissatisfied ☐ Neither satisfied nor dissatisfied ☐ Satisfied ☐ Very satisfied
23. How satisfied are you with the conditions of your living place?
☐ Very dissatisfied ☐ Dissatisfied ☐ Neither satisfied nor dissatisfied ☐ Satisfied ☐ Very satisfied
24. How satisfied are you with your access to health services?
☐ Very dissatisfied ☐ Dissatisfied ☐ Neither satisfied nor dissatisfied ☐ Satisfied ☐ Very satisfied
25. How satisfied are you with your transport?
☐ Very dissatisfied ☐ Dissatisfied ☐ Neither satisfied nor dissatisfied ☐ Satisfied ☐ Very satisfied

26. How often do you have negative feelings such as blue mood, despair, anxiety, depression, etc.?
    ☐ Never ☐ Seldom ☐ Sometimes ☐ Often ☐ Always
27. Do you feel respected or that you have social status?
    ☐ Not at all ☐ A little ☐ A moderate amount ☐ Very much ☐ Completely
28. Are you usually able to eat the foods you want?
    ☐ Never ☐ Seldom ☐ Sometimes ☐ Often ☐ Always
